# Supplementary material for: Bayesian refinement of protein structures and ensembles against SAXS data using molecular dynamics
Source: PLoS Comput Biol. 2017 Oct 18;13(10):e1005800. doi: 10.1371/journal.pcbi.1005800 (PMC5662244; doi:10.1371/journal.pcbi.1005800)
Supplement: S1 Archive — (BZ2) [file pcbi.1005800.s011.tar.bz2 › gromacs_4.62_bayesian_saxs_refi/share/html/online/trjcat.html]

trjcat


| trjcat Main Table of Contents |

**VERSION 4.6  
Sat 19 Jan 2013**

---

### Description

trjcat concatenates several input trajectory files in sorted order.
In case of double time frames the one in the later file is used.
By specifying -settime you will be asked for the start time
of each file. The input files are taken from the command line,
such that a command like trjcat -f \*.trr -o fixed.trr should do
the trick. Using -cat, you can simply paste several files
together without removal of frames with identical time stamps.

One important option is inferred when the output file is amongst the
input files. In that case that particular file will be appended to
which implies you do not need to store double the amount of data.
Obviously the file to append to has to be the one with lowest starting
time since one can only append at the end of a file.

If the -demux option is given, the N trajectories that are
read, are written in another order as specified in the .xvg file.
The .xvg file should contain something like:

0 0 1 2 3 4 5  
2 1 0 2 3 5 4  
Where the first number is the time, and subsequent numbers point to
trajectory indices.
The frames corresponding to the numbers present at the first line
are collected into the output trajectory. If the number of frames in
the trajectory does not match that in the .xvg file then the program
tries to be smart. Beware.

### Files

| option | filename | type | description |
| --- | --- | --- | --- |
| **-f** | traj.xtc | Input, Mult. | Trajectory: xtc trr trj gro g96 pdb cpt |
| **-o** | trajout.xtc | Output, Mult. | Trajectory: xtc trr trj gro g96 pdb |
| **-n** | index.ndx | Input, Opt. | Index file |
| **-demux** | remd.xvg | Input, Opt. | xvgr/xmgr file |

### Other options

| option | type | default | description |
| --- | --- | --- | --- |
| **-[no]h** | bool | no | Print help info and quit || **-[no]version** | bool | no | Print version info and quit || **-nice** | int | 19 | Set the nicelevel || **-tu** | enum | ps | Time unit: fs, ps, ns, us, ms or s || **-xvg** | enum | xmgrace | xvg plot formatting: xmgrace, xmgr or none || **-b** | time | -1 | First time to use (ps) || **-e** | time | -1 | Last time to use (ps) || **-dt** | time | 0 | Only write frame when t MOD dt = first time (ps) || **-prec** | int | 3 | Precision for .xtc and .gro writing in number of decimal places || **-[no]vel** | bool | yes | Read and write velocities if possible || **-[no]settime** | bool | no | Change starting time interactively || **-[no]sort** | bool | yes | Sort trajectory files (not frames) || **-[no]keeplast** | bool | no | Keep overlapping frames at end of trajectory || **-[no]overwrite** | bool | no | Overwrite overlapping frames during appending || **-[no]cat** | bool | no | Do not discard double time frames |

---

http://www.gromacs.org  
gromacs@gromacs.org
